# Tapia’s syndrome: pathogenetic mechanisms, diagnostic management, and proper treatment: a case series

**DOI:** 10.1186/s13256-016-0802-1

**Published:** 2016-01-25

**Authors:** Paolo Cariati, Almudena Cabello, Pablo P. Galvez, Dario Sanchez Lopez, Blas Garcia Medina

**Affiliations:** Maxillofacial Surgery, Hospital Universitario Virgen de las Nieves, carretera de Jaen s/n, 18013 Granada, Andalucia Spain

**Keywords:** Tapia’s syndrome, Endoscopic imagines, Orotracheal Intubation, Diagnostic fibroscopy, Proper treatment

## Abstract

**Background:**

Tapia’s syndrome is an uncommon disease described in 1904 by Antonio Garcia Tapia, a Spanish otolaryngologist. It is characterized by concomitant paralysis of the hypoglossal (XIIth) and pneumogastric (Xth) nerves. Only 69 cases have been described in the literature. Typically, the reported patients presented with a history of orotracheal intubation. Common symptoms are dysphonia, tongue deviation toward the affected side, lingual motility disturbance, and swallowing difficulty.

**Case presentation:**

In the report, we describe three cases of Tapia’s syndrome in three Caucasian patients who underwent surgery with general anesthesia. Two of these patients underwent neck abscess drainage, and the third had an open reduction of a shoulder fracture. The clinical symptoms of Tapia’s syndrome appeared after extubation. All three of our patients recovered their lost function at 3 months after diagnosis.

**Conclusions:**

We underline the importance of performing airway endoscopy and a specific program of swallowing rehabilitation for the proper management of Tapia’s syndrome.

## Background

Tapia’s syndrome is known as a rare complication of airway manipulation. It can occur after any type of surgery that is performed while the patient is under general anesthesia and orotracheally intubated [1]. This syndrome is characterized by neurologic deficits involving the hypoglossal nerve (XII) and recurrent laryngeal branch of the vagal nerve (X). Typical signs and symptoms are dysphonia and swallowing difficulty. The simultaneous emergence of these symptoms in a patient who has undergone orotracheal intubation requires the establishment of a correct diagnostic process to exclude Tapia’s syndrome. Most studies have emphasized that the recovery of nerve function is usually completed within 6 months [2]. However, from our point of view, the establishment of a proper swallowing rehabilitation program is essential to reducing recovery time. In fact, all the patients described in this report were included in a specific rehabilitation program. Each of these programs was coordinated in the dysphagia unit of our hospital. In addition, we highlight that all patients achieved a complete recovery of lost function within 3 months after diagnosis.

## Case presentations

### Patient 1

**Fig. 1:**
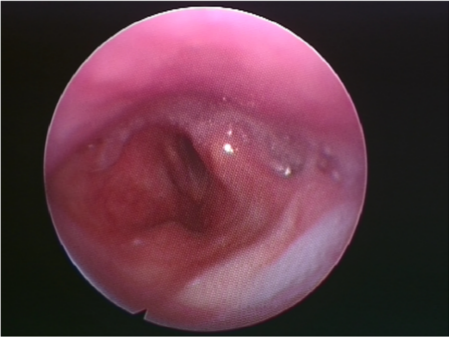
Pyriform sinus retaining saliva

**Fig. 2:**
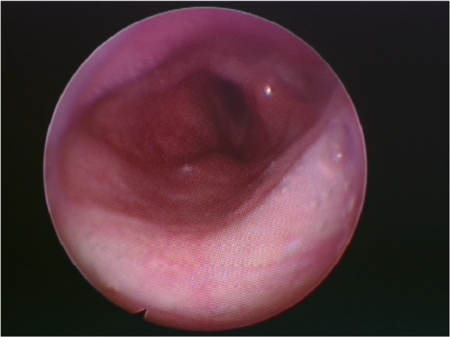
Pharyngoepiglottic muscle hypotonia

A 36-year-old Caucasian man underwent surgical drainage of a neck abscess (dental origin, 47–48) with general anesthesia and orotracheal intubation. A cervical approach was used to reach the submandibular space (right size). The intubation was difficult because of the cervical tumefaction, and it was not possible to perform nasotracheal intubation. The patient woke up with dysphonia, tongue deviation toward the affected side (right size), lingual motility disturbance, and swallowing difficulty. Treatment with barium swallow radiograph excluded the presence of tracheoesophageal fistula. A meticulous neurologic examination and a fibroscopy with swallowing test confirmed nerve paralysis (Figs. 1 and 2).

### Patient 2

**Fig. 3:**
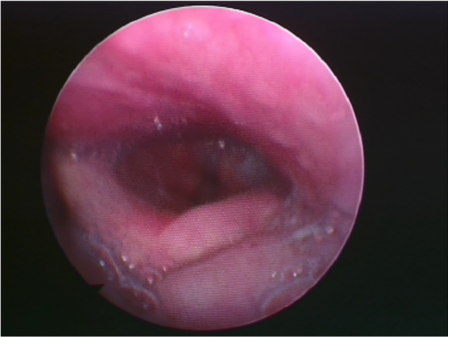
Tongue and epiglottal incompetence

A 61-year-old Caucasian man underwent surgical neck abscess drainage with general anesthesia and orotracheal intubation. A cervical approach to reach the cervical space (right side) was carried out. No problems during the anesthetic procedures were reported. The patient woke up with dysphonia, tongue deviation toward the affected side (right side), lingual motility disturbance, and swallowing difficulty. A meticulous neurologic examination and fibroscopy with a swallowing test confirmed the patient’s nerve paralysis (Fig. 3).

### Patient 3

**Fig. 4:**
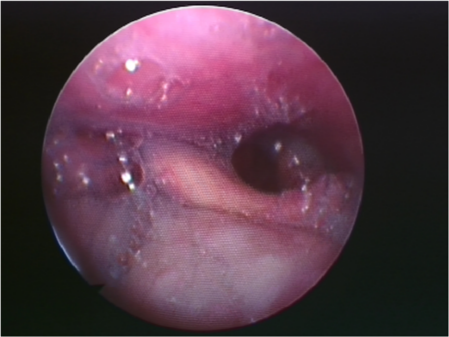
Pyriform sinus retaining saliva

A 42-year-old Caucasian man underwent open reduction of a shoulder fracture with general anesthesia and orotracheal intubation. A direct anterolateral approach to reach the fracture was used. The patient woke up with dysphonia, tongue deviation toward the affected side (right side), lingual motility disturbance, swallowing difficulty, insensibility of the lateral border of the tongue, and lingual bite wounds. An accurate neurologic examination and fibroscopy with a swallowing test confirmed the patient’s nerve paralysis. In this patient, the Xth and XIIth cranial nerve injuries coexisted with paralysis of the lingual branch of trigeminal nerve (Fig. 4).

## Discussion

Tapia’s syndrome is a rare combination of Xth and XIIth cranial nerve palsy. Many of these patients have a clinical history of surgical intervention under general anesthesia and orotracheal intubation. However, different types of surgical intervention could cause Tapia’s syndrome. In fact, the syndrome has been described as a complication of intubation in the intensive care unit [3], after arthroscopic shoulder stabilization [4], and after posterior cervical spine surgery [5]. Specifically, the nerve damage might be caused by the stretching and compressing of the nerves during the orotracheal intubation [6]. Excessive flexion of the head during anesthetic procedures could also be related to the nerve damage [7].

This report raises four central points. First is the correlation between Tapia’s syndrome and anesthetic procedures. In fact, the onset of this disease during the repair of a fractured shoulder reinforces this theory. Second, adequate multidisciplinary support is imperative to ensure a proper diagnosis. Neurology, rehabilitation, and maxillofacial services must work together to make a correct diagnosis and treat the patient optimally. Third, we emphasize that it is essential to perform airway endoscopy to obtain a reliable diagnosis. We think that it is the most useful diagnostic test. In fact, the combination of a meticulous clinical examination and airway endoscopy avoids the use of other diagnostic tests, such as magnetic resonance imaging, electromyography, and treatment with barium swallow radiograph. Fourth, prompt establishment of a swallowing rehabilitation program reduces patient recovery time. It is important to note that all patients reported here were treated in collaboration with the dysphagia unit of our hospital. All three patients recovered lost function at 3 months after diagnosis (Table 1).

**Table 1:** Differences between our patients and other patients reported in the literature

	Our three patients	Cases previously reported in the literature
Diagnostic airway endoscopy	Carried out in all patients	None
Recovery time	All patients recovered lost function within 3 months after diagnosis	6 months [2]
Swallowing rehabilitation program	Carried out in all patients	None
Multidisciplinary approach	Neurology, rehabilitation, and maxillofacial services worked together	None
Demonstrable anesthetic trauma	Yes	Yes [2]
MRI used for diagnosis	None	Yes [6]
Nerve palsy confirmed by neurology service of our hospital	Yes	Not reported
Patient recovery confirmed by rehabilitation service of our hospital	Yes	Not Reported
Failure of epiglottis and pyriform sinus confirmed by video endoscopy	Yes	None

*MRI* magnetic resonance imaging

## Conclusions

We strongly believe that a multidisciplinary approach is required for correct management of this pathology. In fact, we emphasize that, in our three patients, the neurology, rehabilitation (dysphagia unit), and maxillofacial services worked together to guarantee proper management. Moreover, we highlight that establishment of a proper swallowing rehabilitation program reduced the patients’ recovery time. In addition, we stress that airway endoscopy can be the most useful diagnostic test in the management of this syndrome. Indeed, it could avoid the performance of more expensive tests. Finally, we suggest that the incidence of this disorder might be underestimated when it occurs in the head and neck regions. In fact, particularly in the mild forms, the symptoms of Tapia’s syndrome can be confused with discomfort triggered by surgery. This is particularly relevant when surgery includes anatomic areas located close to the Xth and XIIth nerve anatomic routes.

## Consent

Written informed consent was obtained from the patients for publication of this case report and any accompanying images. A copy of the written consent is available for review by the Editor-in-Chief of this journal.

## Abbreviations

MRI: magnetic resonance imaging
